# Glycemic Levels and Insulin Use Daily and During Hospital Shifts in Hospitalized Patients With Type 2 Diabetes

**DOI:** 10.1210/jendso/bvaf137

**Published:** 2025-08-28

**Authors:** Mikkel Thor Olsen, Signe Hjejle Jensen, Louise Mathorne Rasmussen, Carina Kirstine Klarskov, Birgitte Lindegaard, Jonas Askø Andersen, Hans Gottlieb, Suzanne Lunding, Kirsten Nørgaard, Ulrik Pedersen-Bjergaard, Katrine Bagge Hansen, Peter Lommer Kristensen

**Affiliations:** Steno Diabetes Center Copenhagen, Herlev 2730, Denmark; Department of Endocrinology and Nephrology, Copenhagen University Hospital—North Zealand, Hilleroed 3400, Denmark; Department of Endocrinology and Nephrology, Copenhagen University Hospital—North Zealand, Hilleroed 3400, Denmark; Department of Endocrinology and Nephrology, Copenhagen University Hospital—North Zealand, Hilleroed 3400, Denmark; Department of Endocrinology and Nephrology, Copenhagen University Hospital—North Zealand, Hilleroed 3400, Denmark; Department of Pulmonary and Infectious and Diseases, Copenhagen University Hospital—North Zealand, Hilleroed 3400, Denmark; Department of Clinical Medicine, Faculty of Health and Medical Sciences, University of Copenhagen, Copenhagen 2200, Denmark; Department of Clinical Medicine, Faculty of Health and Medical Sciences, University of Copenhagen, Copenhagen 2200, Denmark; Department of Orthopaedic Surgery, Copenhagen University Hospital—North Zealand, Hilleroed 3400, Denmark; Department of Orthopaedic Surgery, Herlev-Gentofte Hospital, Herlev 2730, Denmark; Department of Infectious Diseases, Herlev-Gentofte Hospital, Herlev 2730, Denmark; Steno Diabetes Center Copenhagen, Herlev 2730, Denmark; Department of Clinical Medicine, Faculty of Health and Medical Sciences, University of Copenhagen, Copenhagen 2200, Denmark; Department of Endocrinology and Nephrology, Copenhagen University Hospital—North Zealand, Hilleroed 3400, Denmark; Department of Clinical Medicine, Faculty of Health and Medical Sciences, University of Copenhagen, Copenhagen 2200, Denmark; Steno Diabetes Center Copenhagen, Herlev 2730, Denmark; Department of Endocrinology and Nephrology, Copenhagen University Hospital—North Zealand, Hilleroed 3400, Denmark; Department of Clinical Medicine, Faculty of Health and Medical Sciences, University of Copenhagen, Copenhagen 2200, Denmark

**Keywords:** diabetes, hospital, inpatient, continuous glucose monitoring, insulin, glucose

## Abstract

**Objective:**

To characterize glucose levels and insulin use daily and during hospital shifts throughout hospitalization, which might inform treatment planning and improve outcomes.

**Methods:**

This is a post hoc analysis from a 2-center randomized trial with 166 nonintensive care unit hospitalized patients with type 2 diabetes. Diabetes management was performed by regular staff, guided by diabetes teams using insulin titration algorithms based on either point-of-care glucose testing (POC arm) or continuous glucose monitoring (CGM arm). POC-arm participants wore blinded continuous glucose monitors. The primary outcome was the development in time in range (TIR) (3.9-10.0 mmol/L) between arms during hospitalization.

**Results:**

TIR improved progressively to nearly 90% in the CGM arm by discharge, compared to 60% in the POC arm, which plateaued after day 5 (*P* < .001). Both arms showed the lowest TIR and highest insulin use during the day shift (07:00-15:00 hours). Correctional insulin doses were lower in the CGM arm compared to the POC arm across all shifts: 0.7 IU (±0.3) lower during day shifts (07:00-15:00 hours, *P* = .016), 1.2 IU (±0.4) lower during evening shifts (15:01-23:00 hours, *P* = .005), and 0.3 IU (±0.1) lower during night shifts (23:01-06:59 hours, *P* = .038). Prandial insulin doses were 1.1 IU (±0.5) lower during evening shifts in the CGM arm (*P* = .021).

**Conclusion:**

TIR improved continuously to nearly 90% in the CGM arm by discharge, compared to 60% in the POC arm, which plateaued after day 5, despite lower daily insulin doses in the CGM arm. These findings underscore the sustained effectiveness of CGM in enhancing glycemic levels throughout the entire duration of hospitalization.

Among hospitalized patients, hyperglycemia, hypoglycemia, and high glycemic variability are associated with adverse outcomes, including increased in-hospital mortality, prolonged hospital stays, and higher rates of complications [1-6]. Evidence suggests that correcting dysglycemia can mitigate these risks [7]. Traditionally, in-hospital hyperglycemia has been managed with insulin, guided by point-of-care (POC) glucose testing [8]. However, POC leaves extended periods of time where glucose levels remain unassessed. In response, continuous glucose monitoring (CGM), which automatically measures glucose every 1-5 minutes, has garnered increasing attention for managing hospitalized patients with diabetes [9, 10].

Despite the availability of insulin therapy and glucose monitoring tools, in-hospital diabetes management remains a significant challenge [11]. This is partly due to the limited evidence base supporting inpatient diabetes care, with many guidelines relying on expert consensus rather than robust clinical data [8]. Additionally, glycemic patterns and insulin usage during hospital stays are poorly characterized compared to outpatients [12, 13]. The lack of detailed day-to-day data on glucose levels and insulin use poses a unique challenge in hospital settings, where glucose metabolism can fluctuate rapidly due to various factors, such as medications (eg, glucocorticoids), comorbidities, nutritional therapies, physical inactivity, surgery, infections, and preadmission glycemic status [14, 15]. Managing these dynamic factors requires daily assessment of glucose levels and adjustments in insulin dosing, yet most studies focus on summary metrics of glycemic levels across the entire hospital stay [16]. The latter approach overlooks important day-to-day variations in glucose levels and insulin use, which are critical for good inpatient diabetes care [17, 18].

A detailed characterization of daily glycemic levels and insulin use over the course of hospitalization and across different hospital shifts has not been explored in nonintensive care unit (non-ICU) patients with type 2 diabetes monitored by either POC or CGM. The precise timing of CGM's impact on glycemic levels during hospitalization remains unclear: Does CGM exert a strong influence on glycemic management during the early, middle, or late stages of a hospital stay, or does it consistently provide benefits throughout hospitalization? Understanding these dynamics could guide treatment decisions, highlight critical periods affecting glucose metabolism and insulin management, and inform the cost-effective implementation of CGM in hospital settings.

The objective of this study is 2-fold: (1) to assess glucose levels and insulin usage on a day-to-day basis throughout a hospital stay and (2) to examine these factors during hospital shifts in medical and orthopaedic non-ICU patients with type 2 diabetes, monitored by either POC glucose testing or CGM.

## Materials and Methods

This is a post hoc analysis from DIAbetes TEam and Cgm (DIATEC) trial data (n = 166) [10]. The analyses in the present article are prespecified in the DIATEC trial protocol [19]. The DIATEC trial is a 2-armed, 2-site, prospective, randomized, open-label, blinded-endpoint trial conducted at Copenhagen University Hospital—North Zealand and Herlev-Gentofte Hospital, Denmark. The main findings from the DIATEC trial showed that when implementing CGM alongside a titration algorithm and inpatient diabetes teams, time in range (TIR; 3.9-10.0 mmol/L) increased by 15% points during the entire hospitalization. CGM also significantly lowered time above and below range, glycemic variability, hypoglycemic events, insulin usage, and in-hospital complications during the entire hospitalization [10]. However, day-to-day differences and differences during hospital shifts in glycemic status and insulin usage remain unexplored in patients monitored by either POC glucose testing or CGM.

### Trial Population

We included non-ICU patients from medical and orthopaedic departments with type 2 diabetes aged ≥18 years old and an expected length of hospital stay of at least 2 days after enrolment. Key exclusion criteria included patients who at admission were treated with basal insulin with a duration of action >24 hours (insulin glargine 300 U/mL or insulin degludec), systemic glucocorticoid treatment with prednisone equivalent dose >5 mg/day, and patients in dialysis or with an estimated glomerular filtration rate <15 mL/min/1,73 m^2 ^. Additional eligibility criteria are described in the protocol [19].

### Randomization

An independent statistician not involved in other parts of the trial set up a block randomization list to aim for an equal distribution of patients between the sites and between the POC arm (n = 82) and the CGM arm (n = 84).

### In-hospital Diabetes Management

#### General considerations for patients in the POC arm or CGM arm

In-hospital diabetes management followed an insulin titration protocol [19, 20]. Decisions on insulin titration were based on either POC (POC-arm patients) or CGM (CGM-arm patients). Briefly, on admission, noninsulin antidiabetics were paused, and patients were treated with basal insulin (0.20-0.25 U/kg/day) and correctional short-acting insulin (sliding scale insulin). Prandial insulin (0.20-0.25 U/kg/day) divided into 3 main meals was added at the discretion of the in-hospital diabetes teams. Basal insulin doses were increased if nocturnal hyperglycemia was persistently observed and vice versa. Prandial and correctional insulin doses were initiated/increased if daytime hyperglycemia was persistently observed and vice versa. A detailed description of both the POC-based and CGM-based insulin titration protocol has already been published [19, 20].

#### Treatment in the POC-arm patients only

Subjects in the POC arm were monitored only by POC, and diabetes management was done by usual staff guided by in-hospital diabetes teams. A blinded CGM (Dexcom G6, Dexcom Inc., San Diego, USA) was mounted for outcome analyses.

#### Treatment in the CGM-arm patients only

Subjects in the CGM arm were monitored by POC *and* Dexcom G6 CGM, with real-time CGM data accessible only to the in-hospital diabetes teams. Real-time hypoglycemic (<3.9 mmol/L for 0 minutes) and hyperglycemic (>13.9 mmol/L for 120 minutes) audible CGM alarms were turned on and displayed on tablets in the in-hospital diabetes teams' nurse stations and on transportable smartphones.

### Outcomes

#### Primary outcome

The primary outcome was the development on a day-to-day basis between the POC and the CGM arm in TIR (3.9-10.0 mmol/L) for the first 10 days of hospitalization.

#### Secondary glycemic outcomes

We assessed the daily difference for the first 10 days of hospitalization and differences during hospital shifts (day shifts 07:00-15:00 hours; evening shifts 15:01-23:00 hours; night shifts 23:01-06:59 hours) between the POC and the CGM arm for the outcomes: time above range (TAR) (>10.0 and >13.9 mmol/L); time below range (TBR) (3.0-3.9 mmol/L and <3.0 mmol/L); mean glucose level; SD of the CGM glucose distribution (as a measure of glycemic variability); the coefficient of variation (CV) (defined as SD divided by mean glucose level, as a measure of glycemic variability); total daily doses of insulin; and insulin doses separately for basal, bolus (prandial), and correctional insulin.

### Statistical Analysis

The statistical analyses follow a predefined statistical analysis plan [19] adhering to CGM-based consensus metrics [17]. Glycemic and insulin outcomes for day, evening, and night shifts were compared between the POC and the CGM arm with an independent samples *t*-test or the Mann-Whitney U test according to whether normality assumptions were met or not, respectively. Linear mixed models were used for repeated measures, adjusting for admission day 1 to 10 with an unstructured covariance structure and an interaction effect (*P*-values reported from the interaction term) between time (days 1-10) and allocation (CGM vs POC). Only the first 10 days of hospitalization were included in analyses as the number of patients included on day 11 and onward was close to 0. Data are presented as mean differences (SD) unless otherwise stated. A zero-adjusted gamma distribution accommodates the positivity and exact 0 in the zero-inflated outcome TBR, and data from these analyses are therefore presented as relative differences with a 95% confidence interval (CI). Statistical significance was set at a 2-sided *P*-value ≤.05.

### Ethical Statement

Before enrollment, all subjects provided informed written consent. The Ethics Committee of the Capital Region of Denmark approved the study (journal number 2301240). The trial was prospectively registered with ClinicalTrials.gov (NCT05803473).

## Results

### Baseline Characteristics

Baseline characteristics are published elsewhere [10]. In summary, we included 166 subjects (POC arm, n = 82 and CGM arm, n = 84) with a mean (SD) age of 76.1 (9.8) years, of which 106 (63.9%) were males. The duration of diabetes was on average (SD) 13.1 (8.7) years. In total, 143 (86.1%) patients were on oral antidiabetic medications upon admission, 41 (24.7%) were treated with insulin, 26 (15.7%) were treated with glucagon-like peptide-1 receptor agonists, and 10 (6.0%) were diet regulated solely. The most frequent cause for admission was infection in 60 (36.1%) of patients. Macro- and microvascular diabetes complications were present in 81 (48.8%) and 103 (62.0%) of patients, respectively. Length of hospital stay was median 7.8 (interquartile range 8.7) days. There were no baseline differences between the POC and the CGM arm.

### Glycemic Outcomes

**Figure 1.**
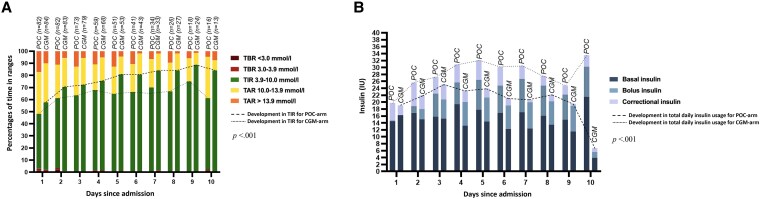
Glycemic levels (A) and insulin usage (B) per day (24 hours) in nonintensive care unit patients with type 2 diabetes monitored by either point-of-care glucose testing or continuous glucose monitoring.

**Figure 2.**
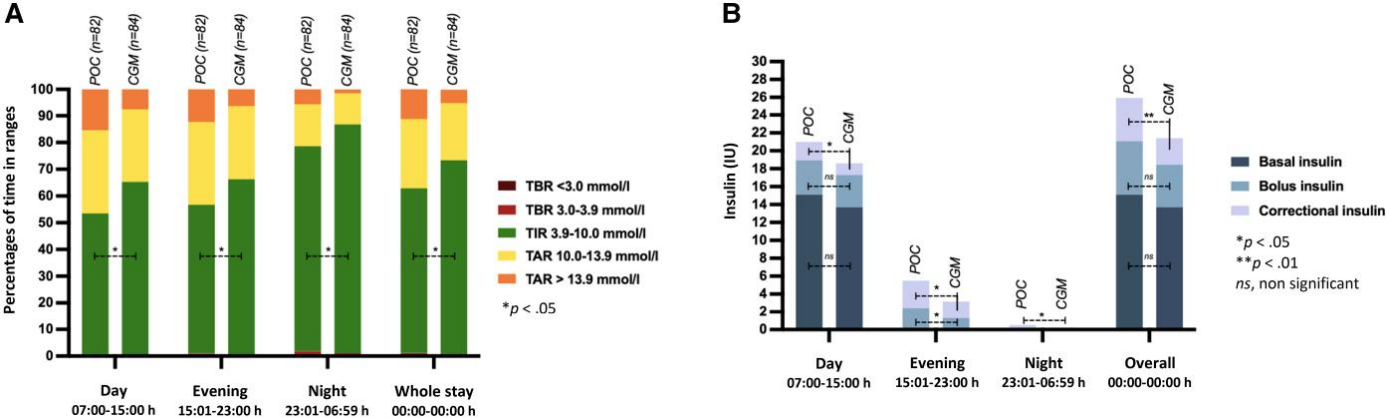
Glycemic levels (A) and insulin usage (B) during day shifts (07:00-15:00 hours), evening shifts (15:01-23:00 hours), and night shifts (23:01-06:59 hours) in nonintensive care unit ill hospitalized patients with type 2 diabetes monitored by either point-of-care glucose testing or continuous glucose monitoring.

**Figure 3.**
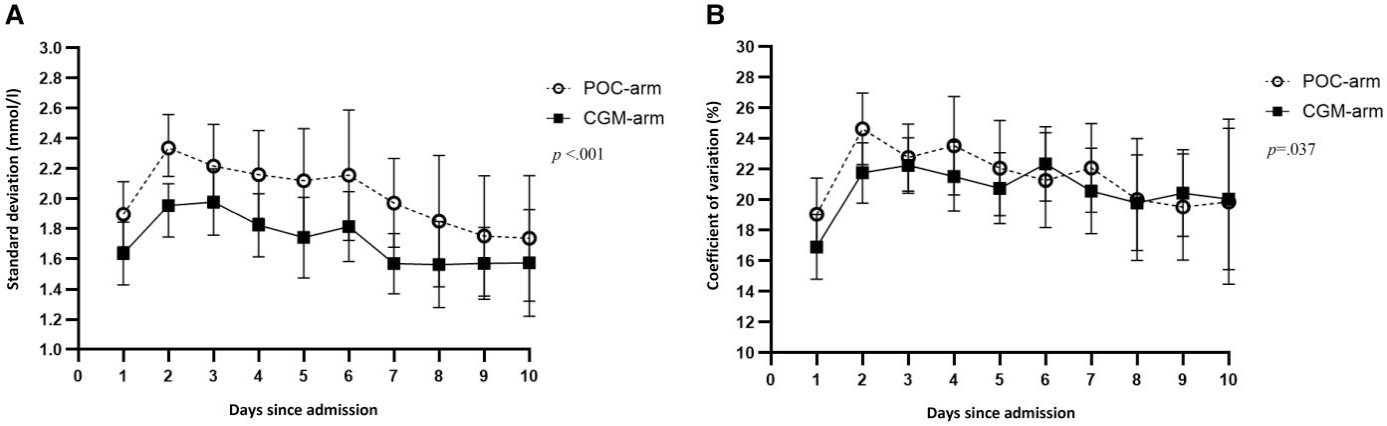
SD (A) and coefficient of variation (B) per day (24 hours) in nonintensive care unit patients with type 2 diabetes monitored by either point-of-care glucose testing or continuous glucose monitoring.

**Figure 4.**
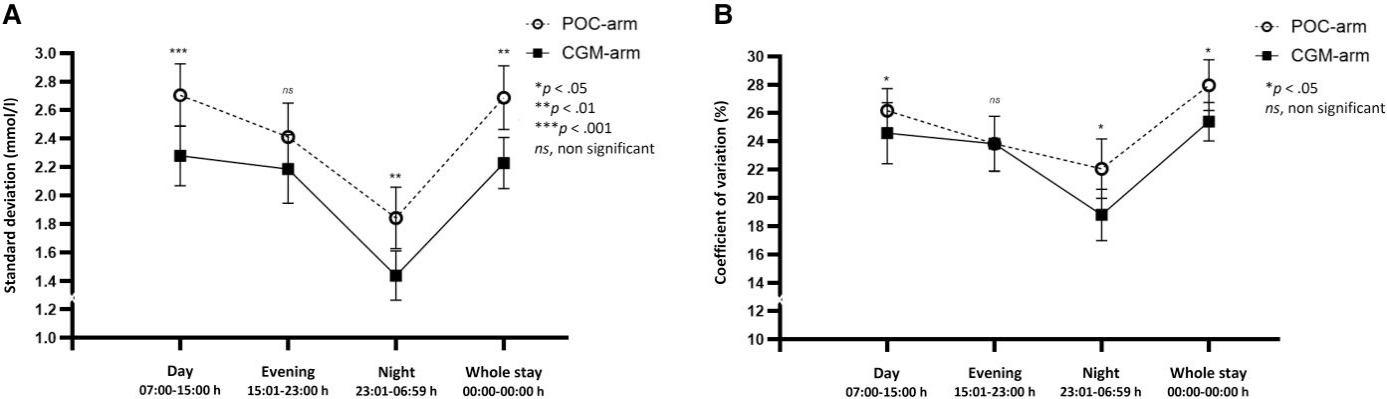
SD (A) and coefficient of variation (B) during day shifts (07:00-15:00 hours), evening shifts (15:01-23:00 hours), and night shifts (23:01-06:59 hours) in nonintensive care unit patients with type 2 diabetes monitored by either point-of-care glucose testing or continuous glucose monitoring.

Glycemic data for TIR, TAR, and TBR per day and per hospital shift are fully presented in Table 1 and visually summarized in Figs. 1A and 2A, respectively, and during the daytime (07:00-23:00 hours) and nighttime (23:01-06:59 hours) each day in Supplemental Fig S. [21]. Glycemic data for SD and CV per day are summarized in Fig. 3A and 3B, respectively, and in Fig. 4A and 4B per shift, respectively.

**Table 1.** Differences in glycemic outcomes between nonintensive care unit hospitalized patients with type 2 diabetes monitored by either point-of-care glucose testing or continuous glucose monitoring

Day/shift	TIR (3.9-10.0 mmol/L) (%)	*P*	TAR (>10.0 mmol/L) (%)	*P*	TAR (>13.9 mmol/L) (%)	*P*	TBR (3.0-3.9 mmol/L) (RD) (95% CI)	*P*	Mean glucose (mmol/L)	*P*	SD (mmol/L)	*P*	CV (%)	*P*
Day 1	10.4 (5.6)	**<**.**001**	9.4 (5.8)	**<**.**001**	−7.2 (3.7)	**<**.**001**	—	—	−0.8 (0.5)	**<**.**001**	−0.3 (0.2)	**<**.**001**	−2.1 (1.6)	.**037**
Day 2	11.8 (4.3)		10.8 (4.4)		−7.0 (2.5)		—		−0.9 (0.4)		−0.4 (0.2)		−2.9 (1.5)	
Day 3	9.1 (4.5)		8.8 (4.5)		−7.1 (2.8)		—		−0.8 (0.4)		−0.2 (0.2)		−0.5 (1.4)	
Day 4	8.0 (5.0)		7.5 (5.0)		−5.6 (2.6)		—		−0.7 (0.4)		−0.3 (0.2)		−2.0 (1.9)	
Day 5	15.4 (5.3)		16.3 (5.3)		−8.2 (3.3)		—		−1.3 (0.5)		−0.4 (0.2)		−1.3 (1.9)	
Day 6	14.1 (5.7)		14.5 (5.7)		−8.8 (2.7)		—		−1.4 (0.5)		−0.3 (0.2)		1.1 (1.9)	
Day 7	12.9 (6.2)		13.7 (6.2)		−4.8 (2.2)		—		−1.1 (0.4)		−0.4 (0.2)		−1.5 (2.0)	
Day 8	17.1 (7.2)		17.2 (7.2)		−7.6 (4.2)		—		−1.4 (0.5)		−0.3 (0.3)		−0.2 (2.5)	
Day 9	13.3 (5.9)		13.6 (5.9)		−4.4 (2.1)		—		−1.1 (0.5)		−0.2 (0.2)		0.9 (2.1)	
Day 10	24.3 (11.1)		23.0 (11.4)		2.7 (6.8)		—		−1.2 (0.9)		−0.2 (0.2)		0.2 (3.4)	
Day shifts	12.0 (4.16)	.**002**	−12.3 (2.9)	.**004**	−8.2 (2.5)	**<**.**001**	0.59 (0.19-1.79)	.**042**	−2.3 (1.4)	.101	−0.5 (0.1)	**<**.**001**	−2.4 (1.0)	.**022**
Evening shifts	10.1 (4.3)	.**010**	−9.7 (3.2)	.**026**	−5.9 (2.4)	.**015**	0.42 (0.13-1.39)	.170	−0.83 (0.3)	.**013**	−0.3 (0.2)	.079	−1.2 (1.2)	.364
Night shifts	9.0 (3.7)	.**008**	−7.9 (3.0)	.**035**	−4.1 (1.7)	.**011**	0.72 (0.26-1.96)	.521	−0.7 (0.3)	.**017**	−0.4 (0.1)	.**004**	−3.3 (1.4)	.**020**

The numbers are presented as the mean differences (SD) between continuous glucose monitoring vs point-of-care unless otherwise stated. *P*-values below .05 are marked in bold.

Abbreviations: CI, confidence interval; CV, coefficient of variation; RD, relative difference; TAR, time above range; TBR, time below range, TIR, time in range.

#### TIR (3.0-10.0 mmol/L)

In summary, TIR increased to nearly 90% in the CGM arm by discharge, compared to 60% in the POC arm, which plateaued after day 5 (*P* < .001) with a sustained benefit of CGM on glycemic outcomes compared to POC across all hospital shifts (*P* < .05). Similar results were observed for day- and nighttime analyses on a day-to-day basis (Fig. S [21]).

#### TAR (>10.0 mmol/L)

The CGM arm consistently showed lower TAR compared to the POC arm on days 1 to 10 (*P* < .001). The same pattern was observed between day, evening, and night shifts.

#### TAR (>13.9 mmol/L)

CGM-arm patients demonstrated consistently lower TAR on days 1 to 10 (*P* < .001). Notable improvements were observed in all hospital shifts, with the greatest difference in dayshift TAR [8.2% points (2.5) lower, *P* < .001], followed by evening and night shifts of 5.9% points (2.4) and 4.1% points (1.7) lower (*P* = .015 and *P* = .011, respectively).

#### TBR (3.0-3.9 mmol/L)

CGM-arm patients' TBR (3.0-3.9 mmol/L) was reduced by a relative difference of 0.59 (0.19-1.79) during day shifts compared to POC-arm patients (*P* = .042). A nonstatistical difference was observed with a relative difference of 0.42 (0.13-1.39) during evening shifts (*P* = .170) and a relative difference of 0.72 (0.26-1.96) during night shifts (*P* = .521). TBR (3.0-3.9 mmol/L) and TBR (<3.0 mmol/L) on a day-to-day basis could not be modelled due to the infrequency of hypoglycemia in this study population.

#### Mean glucose

CGM-arm patients had lower mean glucose on days 1 to 10 (*P* < .001). During day shifts, CGM patients had a nonstatistically significant lower mean glucose of 2.3 mmol/L (1.4; *P* = .101), while evening and night shifts had reductions of 0.83 mmol/L (0.3; *P* = .013) and 0.7 mmol/L (0.3; *P* = .017), respectively.

#### SD

CGM-arm patients had lower SD on days 1 to 10 (*P* < .001). During day shifts, SD was significantly lower by 0.5 mmol/L (0.1; *P* < .001) and during night shifts by 0.4 mmol/L (0.1; *P* = .004).

#### CV

CGM-arm patients had lower CV on days 1 to 10 (*P* < .037) and significant reductions during day shifts of 2.4% points (1.0; *P* = .022) and night shifts of 3.3% points (1.4; *P* = .020).

### Insulin Outcomes

Insulin data for total daily doses of insulin and insulin doses separately for basal, bolus, and correctional insulin per day and shift are fully presented in Table 2 and visually summarized in Figs. 1B and 2B, respectively.

**Table 2.** Differences in insulin usage between nonintensive care unit hospitalized patients with type 2 diabetes monitored by either point-of-care glucose testing or continuous glucose monitoring

Day/shift	Total daily insulin (IU)	*P*	Basal Insulin (IU)	*P*	Bolus (prandial) insulin (IU)	*P*	Correctional insulin (IU)	*P*
Day 1	−0.4 (1.6)	**<**.**001**	1.8 (1.3)	**<**.**001**	−0.2 (0.2)	.182	−2.2 (0.8)	**<**.**001**
Day 2	−3.6 (2.7)		−1.9 (2.1)		1.1 (1.2)		−2.9 (1.1)	
Day 3	−0.9 (3.3)		−0.6 (2.4)		−1.0 (1.8)		−0.7 (1.0)	
Day 4	−6.3 (3.5)		−6.2 (2.4)		0.6 (2.0)		−1.9 (1.2)	
Day 5	−6.6 (4.5)		−3.5 (3.0)		−1.6 (2.3)		−3.1 (1.3)	
Day 6	−7.8 (3.6)		−4.6 (2.7)		−1.1 (2.1)		−3.3 (1.2)	
Day 7	−7.9 (4.9)		−4.7 (3.1)		−2.1 (3.0)		−3.0 (1.1)	
Day 8	−3.1 (5.3)		−2.6 (4.0)		−1.9 (2.7)		−0.9 (0.9)	
Day 9	−4.4 (5.7)		−3.4 (4.3)		0.2 (2.4)		−2.0 (0.9)	
Day 10	−23.8 (5.8)		−17.6 (4.9)		−7.1 (2.0)		−2.3 (1.3)	
Day shifts	−2.5 (1.8)	.166	−2.2 (1.4)	.130	−0.3 (0.7)	.717	−0.7 (0.3)	.**016**
Evening shifts	−1.4 (0.6)	.**013**	No insulin given	—	−1.1 (0.5)	.**021**	−1.2 (0.4)	.**005**
Night shifts	−0.3 (0.1)	.**039**	No insulin given	—	No insulin given	—	−0.3 (0.1)	.**038**

The numbers are presented as the mean differences (SD) between continuous glucose monitoring and point-of-care. *P*-values below .05 are marked in bold.

Abbreviation: IU, international units.

#### Total daily insulin

CGM-arm patients had a lower total daily insulin dose than POC-arm patients of around 4 to 5 units per day on days 1 to10 (*P* < .001). During hospital shifts, insulin was lower in CGM patients with significant differences of 1.4 IU (0.6) during evening shifts (*P* = .013) and 0.3 IU (0.1) during night shifts (*P* = .039).

#### Basal insulin

CGM-arm patients had lower basal insulin doses on days 1 to 10 (*P* < .001). CGM-arm patients' basal insulin dose was 2.2 IU (1.4) lower than POC-arm patients' during day shifts (*P* = .130). All basal insulin was given during day shifts per protocol [19].

#### Bolus (prandial) insulin

No overall differences in bolus insulin were observed on days 1 to 10 between CGM- vs POC-monitored patients (*P* = .182). A significant difference was observed during evening shifts where CGM-arm participants received 1.1 IU (0.5) less bolus insulin (*P* = .021), while day shifts showed no significant differences (*P* = .717).

#### Correctional insulin

CGM-arm patients used less correctional insulin compared to POC-arm patients on days 1 to 10 (*P* < .001). During hospital shifts, the CGM group used lower correctional insulin across all times: day 0.7 IU (0.3; *P* = .016), evening 1.2 IU (0.4; *P* = .005), and night shifts 0.3 IU (0.1; *P* = .038).

## Discussion

In this study, we assessed the differences in glycemic status and insulin doses between non-ICU patients with type 2 diabetes monitored by POC or CGM during hospitalization on a day-to-day basis and during day, evening, and night shifts.

Our findings indicate that patients monitored by CGM experienced more rapid and sustained improvements in TIR and other glycemic parameters compared to the POC glucose testing, largely due to a reduction in TAR (Fig. 1A). In POC-monitored patients, TIR stabilized at around 60% after day 5 of hospitalization, with no further improvement. This aligns with a previous study in 2.759 ICU patients, which reported stable mean glucose levels between days 1 and 7 for those monitored by POC glucose testing [22], although ICU vs non-ICU populations should be compared cautiously. In contrast, CGM-monitored patients in our study showed continuous daily improvement, reaching a TIR close to 90% by discharge, reductions in TAR and mean glucose levels, and a trend toward lower glycemic variability. These results suggest that CGM provides a continuous day-to-day benefit in improving glycemic levels compared to POC glucose testing (Fig. 1A). The improved glycemic levels in both POC- and CGM-monitored patients as the hospitalization progresses likely reflects both the resolution of the underlying illness, reducing stress-induced hyperglycemia, and the optimization of diabetes management during hospitalization, the latter more pronounced in CGM-monitored patients. A previous study has shown that the benefits of in-hospital CGM over POC glucose testing apply universally to all patients with type 2 diabetes, with no specific subgroups showing greater benefit than others [23].

Daily correctional insulin usage was significantly lower in CGM-monitored patients compared to POC-monitored patients. This reduction in correctional insulin doses suggests that basal and prandial insulin dosing was more appropriately adjusted in CGM-monitored patients compared to POC-monitored patients. The highest levels of dysglycemia, characterized by low TIR and high TAR, were observed during day shifts (Fig. 2A), coinciding with the greatest insulin usage (Fig. 2B). Both the POC- and CGM-monitored patients exhibited an inverse U-shaped curve in total daily insulin doses (Fig. 1B). This pattern supports clinical observations that patients typically require increasing insulin doses during the initial days of hospitalization due to altered glucose metabolism—partly influenced by the underlying conditions necessitating their admission—followed by a gradual decrease in insulin requirements toward discharge.

Previous studies investigating CGM in an inpatient setting have shown no or very limited effects of CGM [24]. Two randomized trials were analyzed prematurely due to their suspension caused by COVID-19 [25, 26]. A recent review from 2025, which included 979 patients from 6 randiomized controlled trials on non-ICU patients with diabetes, found that CGM, compared to POC glucose testing, resulted in better glycemic levels and fewer hypoglycemic episodes. Specifically, CGM improved TIR (mean difference 7.2%, 95% CI: 5.1-9.4, *P* < .001), reduced TBR 3.9 mmol/L (mean difference 1.2%, 95% CI: 0.2-2.3, *P* = .02), and decreased TAR 13.9 mmol/L (mean difference 3.7%, 95% CI: 1.3-6.1, *P* = .003). However, no significant differences were observed in glycemic variability or insulin doses between patients monitored with CGM and those using POC glucose testing [10, 27]. Two additional recent trials, the TIGHT trial [28] and a trial by Thabit et al [29], did not observe significant glycemic effects of in-hospital CGM compared to POC.

Experts have suggested that the absence of additional benefits from CGM in hospital settings may be attributed to inadequately trained staff as well as the absence of CGM-based insulin titration algorithms guiding the effective use of CGM data [9, 17, 30-35]. Many of the existing studies discussed previously provide only general guidance on insulin adjustment or fail to present specific protocols for titration based on CGM readings. In contrast, the DIATEC study supports the hypothesis that the effective use of CGM data—through inpatient diabetes teams and operational algorithms [19, 20]—is essential for achieving significant improvements in glycemic control and clinical outcomes, compared to POC glucose testing in the hospital setting. However, before CGM can be broadly implemented in hospital settings, more evidence is required regarding the glycemic effects of in-hospital CGM and optimal implementation strategies to achieve glycemic benefits over POC [36].

Historically, the usage of CGM for inpatient diabetes management has raised concerns about insulin stacking due to the all-time available glycemic data and subsequent hypoglycemia [32] or a state of clinical inertia due to a lack of operational algorithms on how to act on CGM data [35]. Our data suggest that CGM is useful for diabetes management from day 1 and onward with no apparent inertia or indifference to the CGM data occurring close to discharge—even during moderate to long hospitalizations—as reflected in a continuous rise in TIR throughout the stay in the CGM arm. However, it is important to note that this continuous effect of CGM on glycemic outcomes might only be achievable by using CGM-specific insulin titration algorithms and diabetes-educated staff (eg, in-hospital diabetes teams) to actively act on CGM data, avoiding clinical inertia [37].

## Strengths and Limitations

In a 2-center design, we included medical and orthopedic non-ICU patients with type 2 diabetes, increasing the external validity of our findings. Our study aligns with international consensus on in-hospital CGM usage, strongly recommending a protocol to titrate insulin based on CGM data, including alarms [35]. Our study has limitations, including a lack of recruitment of patients receiving nutritional therapies or glucocorticoids during hospitalization, all populations at high risk of developing dysglycemia [38].

## Conclusion

CGM during hospitalization significantly increased TIR, reaching nearly 90% by discharge in non-ICU medical and orthopedic patients with type 2 diabetes. In contrast, TIR for POC-monitored patients tended to stabilize at approximately 60% by day 5 of hospitalization, showing no further improvement thereafter. These findings underscore the sustained effectiveness of CGM in enhancing glycemic levels throughout the entire hospitalization. The highest levels of dysglycemia and insulin usage were observed during day shifts (07:00-15:00 hours). Moreover, daily correctional insulin usage was consistently lower in CGM-monitored patients compared to POC-monitored patients, indicating more proactive and personalized use of prandial and basal insulin in CGM-monitored patients.

## Data Availability

The datasets are available from the corresponding author upon reasonable request.
